# Quercetin shortened survival of radio-resistant B-1 cells *in vitro* and *in vivo* by restoring miR15a/16 expression

**DOI:** 10.18632/oncotarget.27883

**Published:** 2021-02-16

**Authors:** Yasmim Alefe Leuzzi Ramos, Olivia Fonseca Souza, Marilia Campos Tavares Novo, Caroline Ferreira Cunha Guimarães, Ana Flavia Popi

**Affiliations:** ^1^Laboratory of Ontogeny of Lymphocytes, Discipline of Immunology, Departament of Microbiology, Immunology and Parasitology, Universidade Federal de São Paulo, Escola Paulista de Medicina, São Paulo, Brazil

**Keywords:** B-1 cells, chronic lymphocytic leukemia, quercetin, Wnt, miRNA

## Abstract

Chronic lymphocytic leukemia (CLL) is a malignancy disease characterized by the expansion of CD5^+^ B-1 cells. The NZB mouse model of CLL shows similarities to human CLL, has age-associated increase in malignant B-1 clones and decreased expression of miR-15a/16. It was demonstrated that systemic lentiviral delivery of miR-15a/16 ameliorates disease manifestations in this mouse model. Nowadays, new therapeutic approaches have been focus on miRNA in cancer cells. Natural compounds like quercetin can modulate these miRNAs, consequently, suppress oncogenes or stimulate tumor suppressor genes by altering miRNA expressions. Here we investigate the effects of quercetin on miRNA15a/16 expression by radio-resistant B-1 cells. It has been described that a small percentage of B-1 cell survives to irradiation *in vitro*, and these cells show similarities to B-CLL cells. In these cells, the level of miR15a/16 is diminished and Bcl-2 is overexpressed. Quercetin treatment restore both, miR15a/16 and Bcl-2, to normal levels. Furthermore, transference of radioresistant B-1 cells to NOD/SCID mice causes an expansion of this population and also a migration to the liver. However, after quercetin treatment, even radioresistant B-1 cells are not able to expand or disseminate *in vivo*, and the levels of miR15a/16 and Bcl-2 are also normalized. Our data support the hypothesis that quercetin is an important adjuvant molecule that acts on miRNA15a/16 level and leads cells more permissive to apoptosis. This work could help to design new approaches to therapy in CLL patients.

## INTRODUCTION

Chronic lymphocytic leukemia (CLL), the most common leukemia in the Western world, is clinically defined by the accumulation of dysfunctional CD5+ B cells [1]. Heterogeneity of mutations can be associated with CLL pathogenesis. Survival and proliferation of CLL cells depend on intrinsic alterations and also microenvironmental signals [2, 3]. However, it is a not solved puzzle of how the perturbations between signals that promote proliferation and apoptosis could cause the clonal expansion of CD5^+^ B cells.

Several signaling pathways are deregulated in CLL. Wnt/beta-catenin pathway regulates hematopoiesis by controlling the survival, proliferation and differentiation of hematopoietic cells [4]. Consequently, disturbances in the Wnt signaling pathway have an important role in many hematopoietic system malfunctions. Mangolini M et al. [5] show microenvironment-dependent mechanisms of Wnt activation in malignant B cells [5]. Pharmacological inhibition of the Wnt pathway impairs the microenvironment-mediated survival of tumor cells. In comparison to normal B and T cells, B-CLL cells show elevated levels of Wnt3 gene expression [6]. Lu D et al. [4] also described that at least six Wnt genes are overexpressed in CLL cells (Wnt 3, Wnt5b, Wnt6, Wnt10a, Wnt14, and Wnt16) and also Fzd3 and its co-receptor LRP5 and 6. These authors also show that partial inhibition of Wnt/pathway by R-etodolac shortened the *in vitro* survival of the CLL cells, contrariwise to SB-216763 (GSK-3β inhibitor) treatment that enhances CLL survival. However, the translation of this to *in vivo* application was unsuccessful due to its high doses greater than 250 μM. Other small molecule inhibitors (PKF115-584 and CGP049090) of Wnt/beta-catenin pathway succeed to diminish both *in vitro* and *in vivo* CLL survival [7]. These authors stated that these compounds did not affect health B cells *in vitro* and also are well tolerated at doses that are effective for CLL cell killing *in vivo* in the CLL-like xenograft model in nude mice.

Quercetin (3,3′,4′,5,7-pentahydroxyflavone) is another well described inhibitor of Wnt/beta-catenin pathway. It is a natural flavonoid widely present in fruits and beverages [8]. The flavonoid anticancer activity is correlated to its ability to induce apoptosis in tumor cells by increase pro-apoptotic proteins, such as BAX and caspase families, and decrease of the antiapoptotic proteins like Bcl2 [9]. In association with ABT-737, quercetin synergistically induced apoptosis in B-CLL cells [10]. It is also been described that quercetin functional mechanisms are its influence in different miRNA expression [11]. Sonoki et al. [12] demonstrated that quercetin increased miR-16 expression dose-dependently in lung A549 adenocarcinoma cells.

Interestingly, quercetin shortened murine B-1 cell survival *in vitro* and also diminishes the proliferation of these cells [13]. B-1 cells are a subtype of B lymphocytes, that are originate from a fetal precursor and are maintained in adult life mainly by self-renewal of mature B-1 cells. The proliferation and maintenance of B-1 cells are related to constitutively elevated levels of STAT-3, and IL-6 and IL-10 are important cytokines in this process [14]. Novo et al. [13] suggested that reduction in B-1 cell survival after quercetin treatment could be due to a decrease in IL-6 levels in the cultures. B-1 cells express the main Fzd receptors and LRP5 and 6 co-receptors, with high expression of Fzd6 and Fzd9. Wnt3 stimulates B-1 cells to proliferate *in vitro* and also increases the expression of IL7R. Furthermore, B-1 cell precursor in the presence of Wnt3a ligand is induced to differentiate into B-1a cells [15].

In an Eμ-TCL1 mouse model of chronic lymphocytic leukemia, it was described that Wnt16, Wnt10a, Fzd1 and Fzd6 are pronounced increased in CD5^+^ B cells. By crossing Eμ-TCL1 mice with Fzd9^−/−^ or Fzd6^−/−^ mice, the authors observed that Fzd6 impacts on the course of leukemogenesis, but not Fzd9^−/−^ [16]. Besides transgenic mouse models, New Zealand Black/White (NZB/NZW) mouse strain is characterized by age-associated CLL-like symptoms such as splenomegaly and CD5^+^ B1 cell hyper-proliferation with aberrant expression of Pax5, Bcl- 2 and Cyclin-D1 among others [17]. NZB/NZW B-1 cells survive for a long time and increase malignant potential [18]. Also, NZB/NZW B-1 cells are radioresistant to high doses (8 Gy) of ionizing *in vitro* and *in vivo* [19]. Otero et al. [20] demonstrated that high doses of irradiation deplete completely B-2 lymphocytes from peritoneal cavity, but not all B-1 cell populations. Corroborating to this, it has been described that a small percentage of healthy B-1 cells survive to irradiation *in vitro* [21, 22]. Furthermore, this selective radio-resistant B-1 cell population has increased in survival and proliferation activity *in vitro* and also presents hyperploid and CLL morphology [21].

Herein, radioresistant B-1 cells were treated with quercetin *in vitro*, which reduces cell survival and proliferation. This study aimed to determine if quercetin could block the anti-apoptotic profile of radioresistant B-1 cells by interfering in miRNA levels and Bcl-2.

## RESULTS

### Radio-resistant B-1 cells survives and proliferates *in vitro*

**Figure 1:**
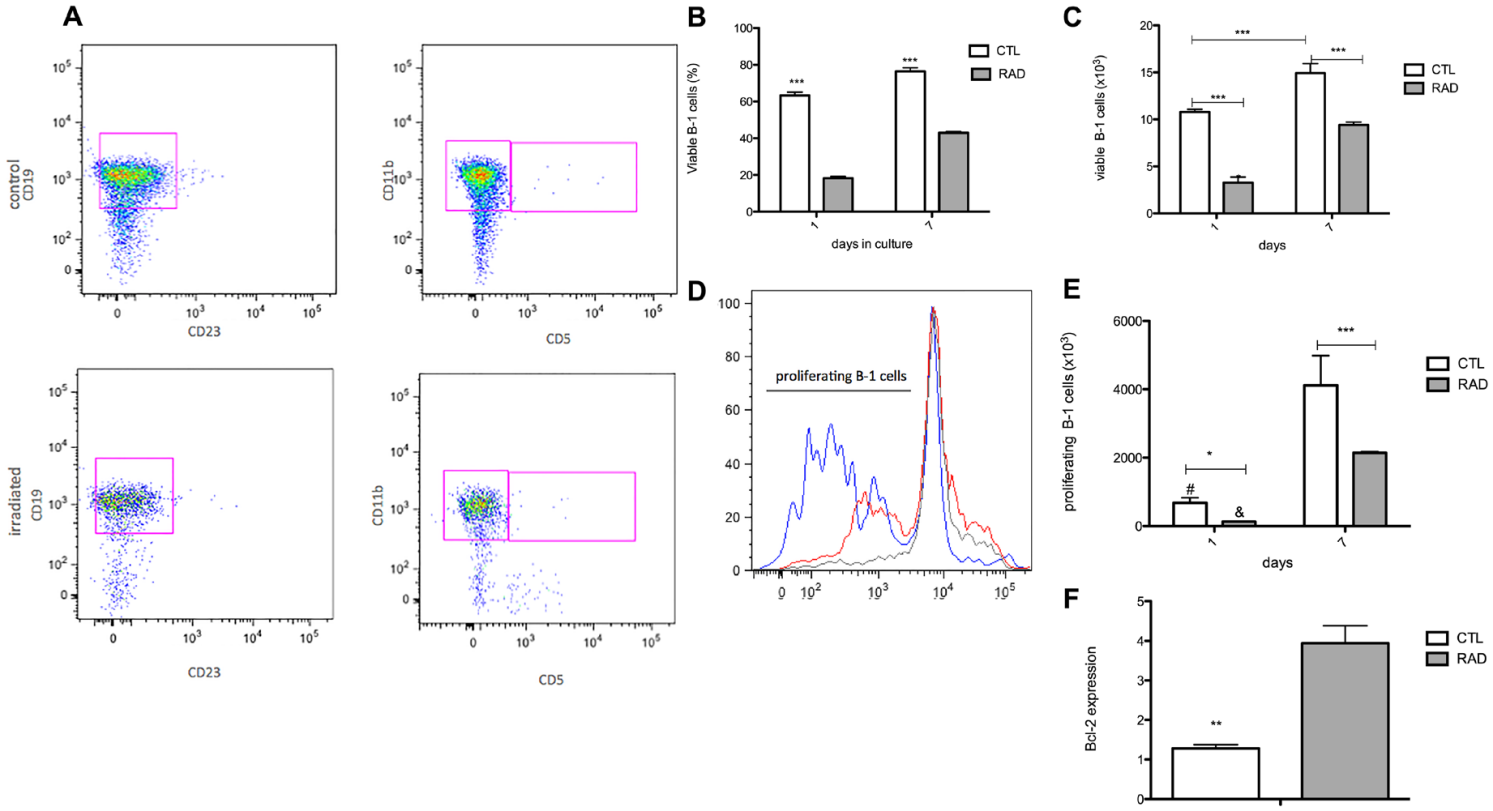
Enlarge of cell survival and proliferation of B-1 cells after irradiation. Peritoneal B-1 cells were submitted to culture after irradiation (3,5Gy–RAD) or not (control group – CTL). These cells were collected and the parameters were analyzed after 1 and 7 days in culture. (**A**) Representative dot plots of B-1 cell phenotype in control and irradiated group, after 7 days in culture. (**B**) Percentage of viable B-1 cells. (**C**) Absolute number of viable B-1 cells. (**D**) Histogram represents the cell proliferation after 7 days in culture. CTL – red, RAD – blue, the cut-off for dye dilution – gray. (**E**) Absolute number of B-1 cells in proliferation. (**F**) Bcl-2 expression by B-1 cells. Data are representative of two independent experiments performed in triplicate. ^*^ *p* < 0,05, ^**^ *p* < 0,01, ^***^ *p* < 0,001, and ^#^ *p* < 0,01 when results in 1 day was compared to same group at 7 days. Student’s *t*-test was performed.

Observed resistance of a small population of B-1 cells to radiation prompted us to investigate the role of miR15a/16 in this phenomenon. Firstly we confirmed that phenotype of B-1 cells and also that part of B-1 cells are able to survive *in vitro*, at least for 7 days after irradiation (Figure 1A–1C). It is important to mention that B-2 cells do not survive *in vitro* after irradiation (data not shown) [20, 23, 24]. It is clear that the number of viable cells after irradiation decreases in relation to control group, however it is important to note that the proliferation of these cells augments after 7 days (Figure 1D–1E). At day 7, it is possible to observed a ~6 fold increase in control group, while irradiated B-1 cell pool increase ~14 fold. This result suggested that despite a large population of B-1 cells has been affected by irradiation, radio-resistant B-1 cells that survive in culture are resistant to apoptosis and also able to proliferate. The apoptosis resistance is corroborated by elevated levels of Bcl-2 expression in radio-resistant B-1 cells (4 fold increased in comparison to control group – Figure 1F).

### Quercetin treatment facilitates apoptosis of radio-resistant B-1 cells

**Figure 2:**
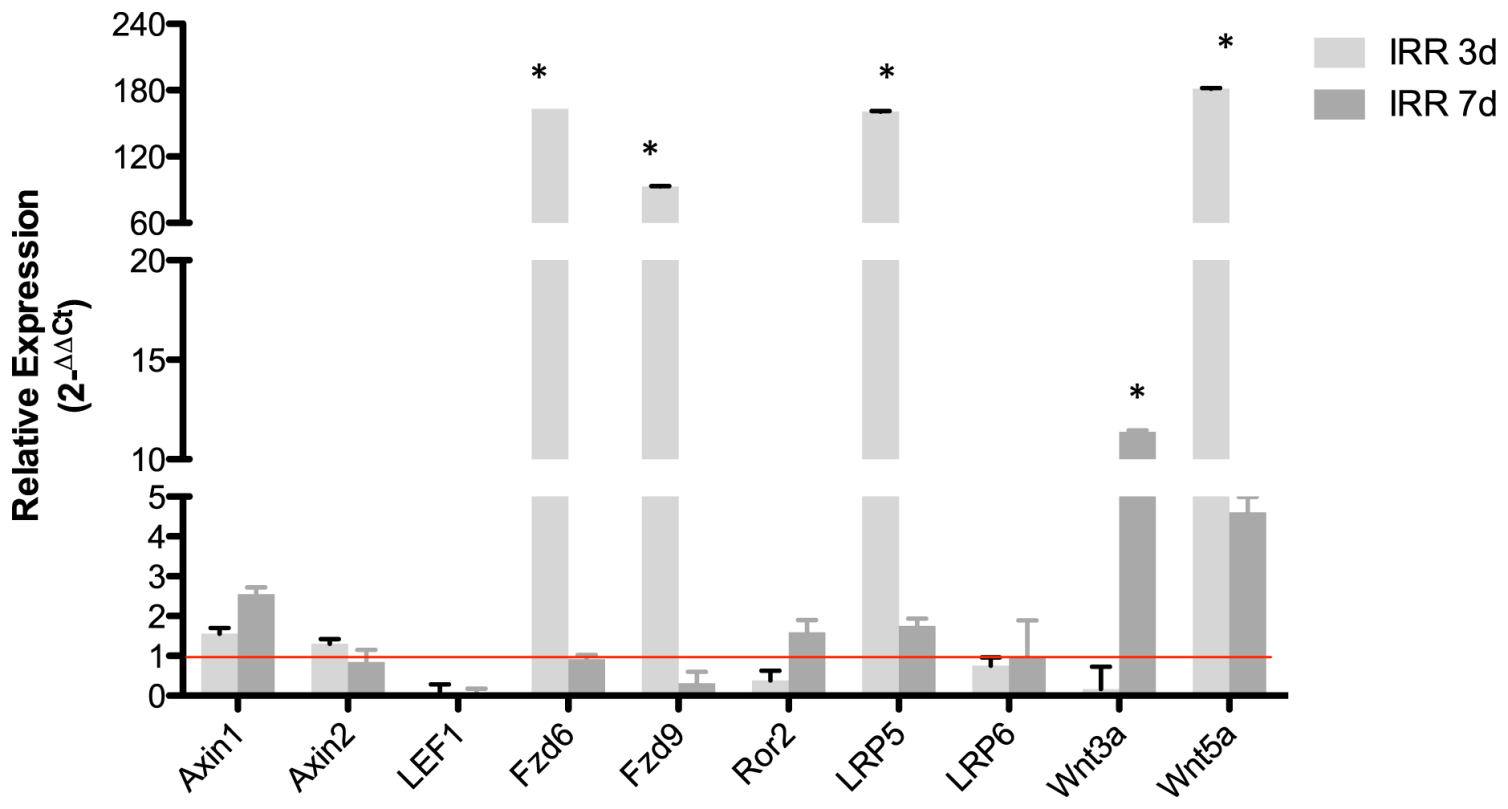
Radio-resistant B-1 cells have higher expression of Wnt3a, Fzd6 and LRP5. Relative expression of some components of Wnt pathway. Expression was normalized by control group (non irradiated cells). Student’s *t*-test was performed. ^*^ *p* < 0,01. All values are statically different from control group, except axin2, Fzd6 (7days), LRP6. Data are representative of two independent experiments performed in triplicate.

Several pathways are involved in lymphocyte proliferation, further it is known that some pathways are involved in the increase proliferation status or resistance of apoptosis in neoplastic cells. Previous work has shown that Wnt/beta-catenin pathway is important to B-1 cell survival *in vitro* [13]. The blockage of Wnt/beta catenin pathway by quercetin reduces B-1 survival and proliferation *in vitro* [13]. To focus on this pathway, it was analyzed the expression of the main components of Wnt/beta-catenin pathway after irradiation. Interestingly, the irradiation increase the expression of the main components of Wnt/beta-catenin signaling in B-1 cells, such as the receptors Fzd6, Fzd9, LRP5 and also the Wnt3a ligands (Figure 2).

**Figure 3:**
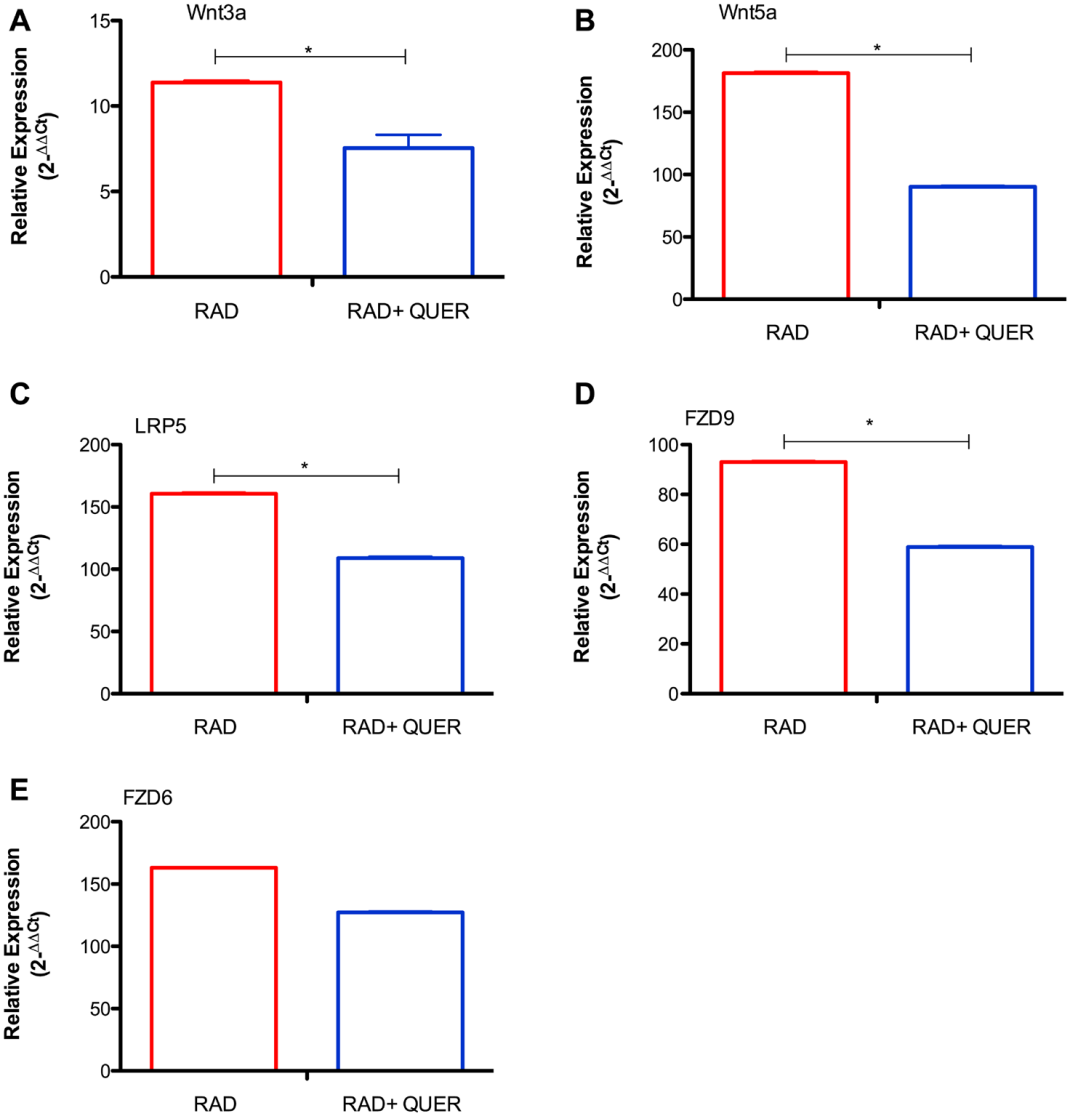
Quercetin treatment reduces expression of Wnt pathway components. Relative expression of Wnt3a (**A**), Wnt5a (**B**), LRP5 (**C**), Fzd9 (**D**) and Fzd6 (**E**). Expression was normalized by control group (non irradiated cells). Student's *t*-test was performed. ^*^ *P* < 0,01. Data are representative of two independent experiments performed in triplicate.

**Figure 4:**
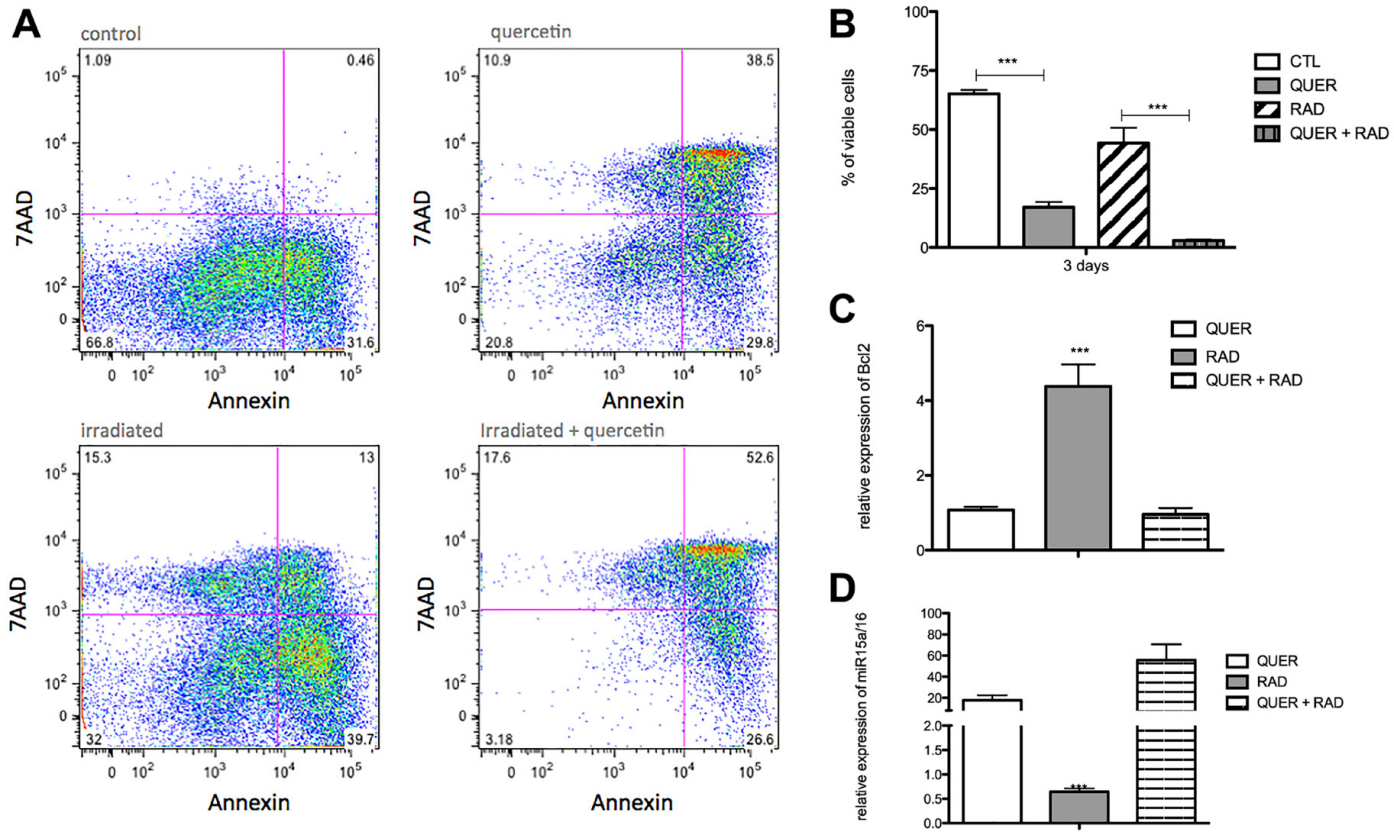
Quercetin treatment reverts the anti-apoptotic effects of irradiation. Peritoneal B-1 cells were submitted or not to irradiation and cultivated for 3 days in the presence or not of quercetin (100 μM). (**A**) Representative dot plot of Annexin V/7-AAD staining of control, quercetin, irradiated and irradiated + quercetin groups. (**B**) Percentage of viable B-1 cells. The cell viability was assessed by Annexin V Apoptosis Detection Kit. (**C**) Relative expression of BCL-2. (**D**) Relative expression of miR15a/16. Control group (non-treated cells) is the normalizer sample. ^*^ *p* < 0,01, ^**^ *p* < 0,001. Data are representative of two independent experiments performed in triplicate. Student's *t*-test or One-Way ANOVA with Bonferroni post hoc test.

Next, quercetin treatment was used to block the Wnt/beta-catenin pathway. In comparison to radiated B-1 cells, the presence of quercetin diminishes levels of Wnt3a, Wnt5a, Fzd9, LRP5 (Figure 3A–3D). Despite of Fzd6 is increase in RAD and RAD+QUER group in relation to control group; it was no observed difference between these two experimental group (Figure 3E). As demonstrated previously [13], quercetin treatment decreases the viability of B-1 cells in culture after 3 days in comparison to control. This effect was also observed in radio-resistant B-1 cells. Addition of quercetin to radio-resistant B-1 cell culture abolishes its resistance to apoptosis (Figure 4A and 4B). Furthermore, quercetin treatment of irradiated B-1 cells provokes a reduction in Bcl-2 expression (Figure 4C). One of mechanism that regulated Bcl-2 expression is miR15/16a [25]. Corroborating the previous result, the levels of miR15a-16 are reduced when B-1 cells were irradiated, but it is increased when these cells are treated with quercetin (Figure 4D). Taking together, these data suggest that quercetin treatment could influence the levels of Bcl-2 and also restore the levels of miR15a-16.

### Quercetin treatment is able to reduce B-1 cell expansion *in vivo*

**Figure 5:**
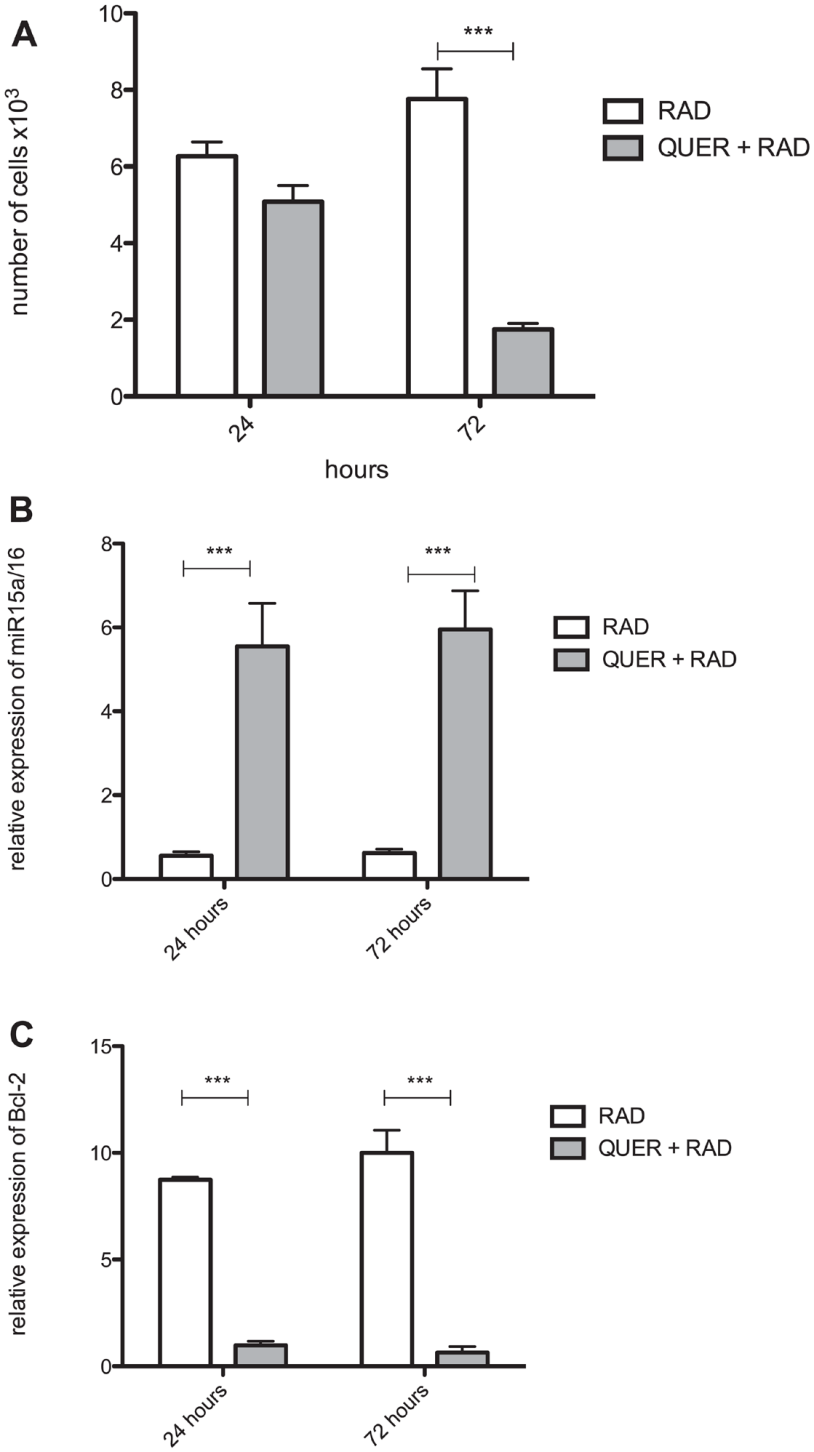
Quercetin blocks survival of radio-resistant B-1 cells *in vivo*. Peritoneal B-1 cells were submitted to irradiation and cultivated for 3 days in the presence or not of quercetin (100 μM). These cells were inoculated i.p. in NOD/SCID mice. After 24 and 72, peritoneal cells were collected and the amount of B-1 cells recovered was analyzed. (**A**) Number of viable B-1 cells. (**B**) Relative expression of miR15a/16. (**C**) Relative expression of BCL-2. In B and C, control group (non-treated B-1 cells) normalized the samples. ^*^ *p* < 0,01, ^*^ *p* < 0,001. Data are representative of two independent experiments performed in triplicate. One-Way ANOVA with Bonferroni post hoc test.

It has been demonstrated that radio-resistant B-1 cells acquire some characteristics of CLL-like cells [21]. Based on previous results, we investigated if irradiated B-1 cells could survive *in vivo*. Radio-resistant B-1 cells and quercetin-treated radio-resistant B-1 cells were adoptively transferred to peritoneal cavity of immunodeficient mice. As observed in Figure 5A, after adoptive transference irradiated B-1 cells are able to survive *in vivo* in the peritoneal cavity. However, when these cells were treated with quercetin the number of cells recovered from peritoneal cavity was at least 3× time decreased (Figure 5A). It was also detected that quercetin treatment restored the levels of miR15a/16 in irradiated B-1 cells (Figure 5B), even after 72 hours of transference *in vivo*. Corroborating to this, it was observed that BCL-2 expression is decreased in irradiated B-1 cells after quercetin treatment (Figure 5C).

**Figure 6:**
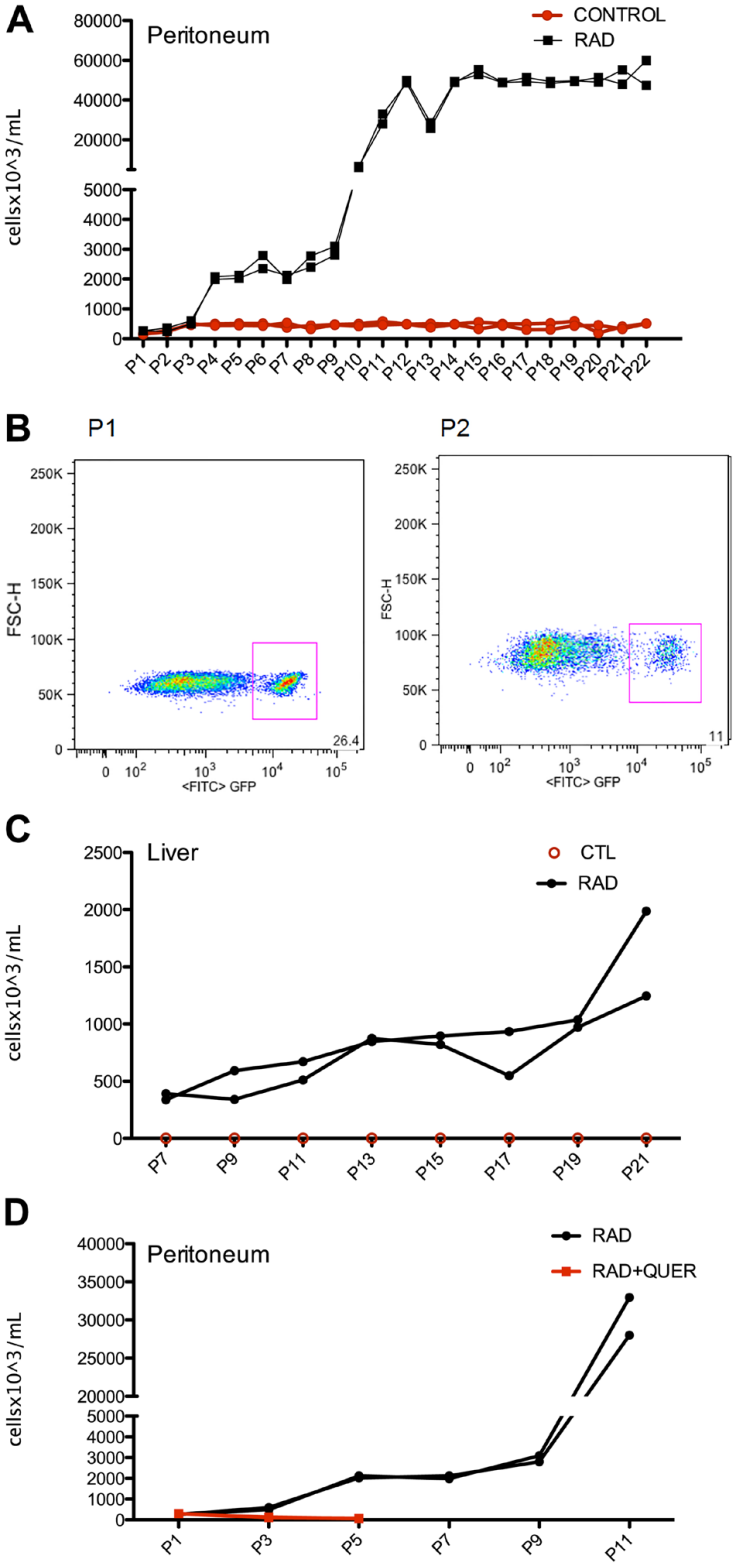
Irradiated B-1 cells survival and disseminate *in vivo* after multiple passages. Peritoneal B-1 cells (GFP^+^) were submitted to irradiation and cultivated for 3 days in the presence or not of quercetin (100 μM). These cells were inoculated i.p. in NOD/SCID mice. After 15 days, peritoneal cells were collected, peritoneal B-1 cells (GFP+) was recovered and inoculated in another mice. This procedure was repeated each 15 days for 22 passages. (**A**) Number of B-1 cells (GFP+) recovered in each passage from peritoneal cavity. (**B**) Representative plots of GFP+ gate from passage 1 (P1) and passage 22 (P22) from peritoneal cavity of mice that received irradiated B-1 cells. (**C**) Number of B-1 cells (GFP+) recovered from liver. It is important to note that only irradiated B-1 cells (GFP+) were found in the liver, B-1 cells from control group were not detected. (**D**) After quercetin treatment, B-1 cells are not able to survive and expand *in vivo*. Number of B-1 cells (GFP+) recovered from peritoneal cavity from mice inoculated with radio-resistant B-1 cells (RAD) or quercetin-treated radio-resistant B-1 cells (QUER+ RAD). *n* = 2 for each experiment.

Besides to survive to adoptive transference, irradiated B-1 cells enlarge and migrate to other organs. To demonstrate that irradiated B-1 cells acquire resistance to apoptosis, multiple passages *in vivo* were made after irradiation. Firstly, B-1 cells were submitted to irradiation and culture for 72 hours. After this, these cells were injected in the peritoneal cavity of immunodeficient mice. After 15 days cells from peritoneum were collected and GFP+ cells are counted (Figure 6B). These cells were separate by cell sorting and inoculated to other mice. As observed in Figure 6A, after 4 passages, despite B-1 cells were recovered from peritoneal cavity, no expansion of B-1 cell population was observed. It is important to mention that B-1 cells from control group (non-irradiated) were also transferred to mice as control, but non expansion was observed along the time, meaning that same amount of cells (~5 × 10^5^ cells) was recovered along the time (Figure 6A). However, after P10 it was observed a huge expansion of irradiated B-1 cells. Furthermore, irradiated B-1 cells were also detected on liver after P7 (Figure 6C). GFP+ cells were not detected in the liver when control B-1 cells were transferred. B-1 cells derived from control group were also not found in bone marrow, peripheral blood and spleen.

It was also observed that quercetin treatment of irradiated B-1 cells abolish the ability to expand *in vivo*, and also to migrate to other organs. In this group only 5 passage were performed because the number of cells recovered in P5 was less than 100 cells per mice (Figure 6D). This result suggest that restoration of miR15a/16 by quercetin treatment could be an important factor to the restitution of apoptosis sensibility of B-1 cells.

## DISCUSSION

One of the challenges against CLL is the purpose of new therapeutic strategies to bypass the acquired resistance to apoptosis cells. Several reports bring evidences that quercetin treatment is able to reduce CLL survival *in vitro* [10, 26–28]. The novelty in the data showed here is that it is clearly demonstrated that quercetin shortened the survival of radioresistant B-1 cells *in vitro* by restored miRNA 15a/16 levels and reduce the expression of BCL-2.

In human CLL, some previous studies suggested that Wnt signaling components are significantly up regulated compared with their expression in normal B cells [4, 29]. Wu et al. [16] demonstrated that up-regulation of Fzd6 is important, although not essential, to leukemogenesis. Corroborating to this, Eμ-TCL1 animals lacking the Fzd6 gene show decreased levels of beta-catenin and delay in tumor growth [16]. Herein, it was demonstrated that radioresistant B-1 cells could be more responsive to Wnt signaling considering that several Wnt components; mainly Fzd6 receptors and co-receptors are higher expressed in these cells in comparison to normal B-1 cells. Besides to this, radioresistant B-1 cells also augment the levels of Wnt3, which could amplify the Wnt signaling and maintain the cell survival.

Antagonizing Wnt signaling in radioresistant B-1 cells with quercetin results in diminish of cell survival *in vitro* and *in vivo*. Corroborating to this we demonstrate that levels of miR15a/16 are restored and Bcl-2 are diminished. It has been described that quercetin directs bind to the BH3 domains of Bcl-2 and Bcl-x proteins, causing inhibition of their anti-apoptotic activity [30]. The mechanisms involved in the augment of antiapoptotic Bcl-2 factors in CLL are still unclear. Some patients carry del13q14, which causes the deletion of microRNAs miR-15a and miR-16, which down-regulate Bcl-2 expression [25, 31]. Liu et al. [32] demonstrated that p53-miR-15a/16-Mcl-1 axis may be a critical pathway in the regulation of apoptosis and drug resistance in CLL, considering the impact of quercetin in decrease the viability of radioresitant B-1 cells by modulate the levels of miR15a/16-Bcl-2 could point an important adjuvant in the CLL treatment.

Interestingly, radioresistant B-1 cells were able to expand *in vivo* and also disseminate to other organs such as liver. Quercetin treatment reverts this phenomenon, because quercetin-treated radioresistant B-1 cells transferred to a peritoneal cavity of mice were not recovered after 72 hours. The levels of miR15a/16 in these cells are augment. Furthermore, when these quercetin-treated radioresistant B-1 cells were submitted to a multiple passage *in vivo*, few cells were recovered and no expansion or dissemination was observed. Corroborating to this, Underbayev et al. [33] demonstrated that both miR-15a deficient HSC and B-1 cell progenitors are capable of repopulating irradiated recipients and produce higher numbers of B1 cells than sources with normal miR-15a/16 levels.

It has been showed that miR15a down-regulates a number of critical genes relate to lymphocyte survival, such as IL10 and Mmp10. Studies with NZB IL10 knockout mice evidence the IL10 role in B-1 cell expansion and development of CLL [33, 34], whereas Mmp10 works by accelerating the tumor growth [35]. Despite of quercetin did not influence IL-10 levels in B-1 cell culture, it was detected high levels of IL-6 after quercetin treatment [13]. Considering all these data together, we could postulate that quercetin acts on miR15a and suggest that it could interferes in several mechanisms of B-1 cell survival.

Alterations in the primary mir-15a/16-1 loci were associated to the development of CLL in the New Zealand Black murine model (14), and exogenous miR-16 levels restored levels of cyclin D1 in B cells from NZB mice. Furthermore, restoration of miR-16 levels enhances the ability of nutlin and genistein to promote apoptosis of malignant B-1 cells [36]. Based on this, quercetin treatment could be an adjuvant to increase the sensibility of malignant cells to therapy.

Several small molecules has been described to inhibit Wnt pathway and for this reason controls CLL growth [7]. In this context the main concern is a usage dose that have more efficiency than deleterious effects. Herein, we demonstrated a non-toxic and prolonged effect of quercetin in control radio-resistant B-1 cells. We postulate that this approach could be important for those patients that have a reduction on miR15a/16 levels, but not ch13q14 deletions. Of course, this study must have to be more detailed before to be extrapolated to human therapy. However it is an important clue of how molecular mechanisms in a malignant cell could be explored in benefit to development of new therapies or improvement of existing ones.

## MATERIALS AND METHODS

### Animals

C57Bl/6, C57Bl/6 GFP and NOD/SCID mice from 6 to 8 weeks of age were obtained from the Centro de Desenvolvimento de Modelos Experimentais para Biologia e Medicina (CEDEME) of the Universidade Federal de São Paulo (UNIFESP, Brazil) and housed under specific pathogen-free conditions at animal facility of the Discipline of Immunology / UNIFESP. This project was approved by the research ethics committee of UNIFESP (CEUA N° 4035240615 and 2014/8832030914).

### Peritoneal B-1 cell culture

Peritoneal cells were obtained and submitted to irradiation (3.5 Gy). Then, these cells were dispensed into 24-well plates (Corning Costar, Tokyo, Japan) and incubated at 37°C in 5% CO2 for 40 min. Non-adherent cells were discarded, and RPMI-1640 containing 10% fetal calf serum (Cultilab, Campinas, SP, Brazil) (R10) was added to the adherent fraction, followed by incubation at 37°C in 5% CO2. Accordingly to Almeida et al. [37], after 24 h B-1 cells are the main cell type in the non-adherent cell populations. Herein, non-adherent cells (B-1 cells) were collected and used in some experiments. Nonirradiated B-1 cells were cultivated in the same conditions and were used as control. Quercetin (100 μM) was added daily to cultures in quercetin-treated group (QUER) and quercetin-treated irradiated group (RAD + QUER).

### Viability analysis

B-1 cells from peritoneal cell culture were collected and resuspended in 1 ml of PBS with 1 ul of the marker LIVE/DEAD Fixable Aqua Dead Cell Stain Kit (Life Technologies) and incubated for 30 min at 4°C. After this, cells were stained with the following antibodies: phycoerythrin (PE) rat anti-mouse CD19, fluorescein-isothiocyanate (FITC) rat anti-mouse CD23 and peridinin chlorophyll protein complex (PerCP) rat anti-mouse CD11b. All antibodies were from BD Biosciences (Pharmingen, San Diego, CA). Cells were maintained for 25 min at 4°C. Fifty thousand events were acquired on Attune^®^Acoustic Focusing Flow Cytometer (Life Technology, Applied Biosystems), and analysis was performed using FlowJo software (Tree Star). For the analysis, CD19+CD23−CD11+CD5+/− cells (B-1 cells) number was determined. Considering this population, viable or non-viable cells were counted based on the LIVE/DEAD staining.

### Apoptosis detection

B-1 cells were cultured in the conditions described previously, collected and stained for cell death using Annexin V Apoptosis Detection Kit (BDPharmingen), accordingly to manufacturer’s instructions. Briefly, cells were resuspended in the Binding Buffer, and 100 μl of cell suspension were used for apoptosis staining. These cells were incubated with 5 μl/sample of PE Annexin-V and 5 μl/sample of 7-AAD for 15 min at room temperature in the dark. After this, 400 μl of Binding Buffer was added and cells were submitted to acquisition by FACSCantoII (BD Biosciences). The analysis was performed using FlowJo software (Tree Star).

### Cell trace violet proliferation assay

B-1 cells were cultured in the conditions described previously and proliferation was measured using Cell Trace Violet Proliferation Kit (Life Technology), accordingly to manufacturer’s instructions. The acquisition was performed in the Attune^®^AcousticFocusing Flow Cytometer (Life Technology, Applied Biosystems) instrument and analysis were performed using FlowJo software (Tree Star).

### B-1 cell enrichment

Cells from the peritoneal B-1 cell culture, submitted to conditions described in each experiment, were submitted to separation by selection in FACSAriaII (BD Biosciences). Cells in the non-adherent fraction were harvested, labeled with biotin-conjugated rat anti-mouse CD19 (BD Biosciences) and the CD19+ population enriched by using Anti-Biotin MicroBeads and MS Columns (Milteny Biotec) accordingly to the manufacturer instructions. Enrichment of ≥ 98% of CD19+ cells was set as the minimum acceptable experimental condition. The percentage of B-1 cell enrichment was verified by the addition of streptavidin-PE (BD Biosciences) and analysis in a FACS CantoII system (BD Biosciences).

### RNA isolation and quantification

Total RNA was extracted using Tryzol (Invitrogen, Carlsbad, CA, USA) according to the manufacturer’s instructions. miRNA specific cDNA was prepared using the TaqMan^®^ MicroRNA Reverse Transcription (Applied Biosystems, Foster City, CA, USA). The following pre-made TaqMan Assays (Applied Biosystems) were used for real-time quantification: mmu-miR-15a-5p (Assay ID 000389), U6 (Assay ID 001973).

Bcl-2, FZD receptors gene, Wnt3a, Wnt5a, AXIN2 transcripts were quantified using the cDNA was obtained using FAST Sybr Green Reagent (Thermo Fisher Scientific, Carlsbad, CA, USA), from cDNA obtained using the Superscript IV cDNA Synthesis (Thermo Fisher Scientific, Baltics, Lithuania). RPLP0 expression was used as a normalization housekeeping control. Relative quantification was determined according to the 2^−ΔΔCt^ method. Each reaction was carried out in triplicate using at least 3 biologic samples. The sample used as normalizer was B-1 control.

### B-1 cell transference

B-1 cells were obtained from peritoneal cavity of C57Bl/6 GFP mice. The culture of peritoneal cells was performed as described before, and cells were submitted to irradiation and/or quercetin treatment. Quercetin (100 μM) was added daily in cultures. After 72 hours, B-1 cells were enriched by cell sorter as described earlier. Approximately, 6 × 10^5^ cells were injected intraperitoneally (i.p.) in NOD/SCID mice. After 24 and 72 hours, peritoneal cells were collected and the amount of GFP+ cells was analyzed. These cells were sorted and submitted to RNA extraction to analyses of miRNA and Bcl2 expression.

For multiple passage experiments, the same protocol above as performed. After 15 days cells from peritoneum were collected and GFP+ cells are counted. These cells were separated by cell sorting and inoculated i. p. to another NOD/SCID mice. A total of 22 passages were performed. The passage was stopped at 22nd in respect of humane endpoint because at this time the animals show a 30–40% weight loss.

## Abbreviations

CLL: Chronic lymphocytic leukemia
NZB/W: New Zealand Black/White mice
NOD/SCID: Nonobese diabetic/severe combined immunodeficiency mice
GFP: green fluorescence protein
